# Physiological stress responses of tigers due to anthropogenic disturbance especially tourism in two central Indian tiger reserves

**DOI:** 10.1093/conphys/coz045

**Published:** 2019-07-12

**Authors:** Abhinav Tyagi, Vinod Kumar, Sagar Kittur, Mahender Reddy, Sergey Naidenko, Andre Ganswindt, Govindhaswamy Umapathy

**Affiliations:** 1 Laboratory for the Conservation of Endangered Species, Council for Scientific and Industrial Research—Centre for Cellular and Molecular Biology, Uppal Road, Hyderabad, Telangana, India; 2 A.N. Severtsov Institute of Ecology and Evolution, Leninsky, pr. 33, Moscow, Russia; 3 Mammal Research Institute, Faculty of Natural and Agricultural Sciences, University of Pretoria, Private Bag X20, Hatfield, South Africa

**Keywords:** Anthropogenic disturbance, Bandhavgarh Tiger Reserve, faecal glucocorticoid metabolites, Kanha Tiger Reserve, stress, tiger, tourism

## Abstract

Tigers continue to face unprecedented threats to their existence due to poaching, habitat loss, habitat fragmentation and anthropogenic disturbances. The present study examines the physiological stress response of tigers due to anthropogenic activities including wildlife tourism in Bandhavgarh Tiger Reserve and Kanha Tiger Reserve using faecal glucocorticoid metabolite (fGCM) measurement. We collected a total of 341 faecal samples from both reserves during tourism and non-tourism periods. Data on various anthropogenic disturbances including tourism activities like number of vehicles and visitors were also collected. We ascertained the species identity and sex of all the samples collected using genetic markers. fGCMs were extracted using a previously reported procedure, and fGCM concentrations were subsequently determined using an established enzyme immunoassay. There was no significant difference in overall mean fGCM concentrations between the two tiger reserves, but within each reserve, concentrations were significantly higher in tigers during the tourism period as compared to the non-tourism period. We also found that the number of tourist vehicles and disturbance level significantly correlated with fGCM concentrations. This study further supports the assumption that unbridled tourism associated with high anthropogenic disturbance can be related to perceived stress and consequently may have an impact on the reproductive fitness of tigers and long-term survival of isolated populations.

## Introduction

Large carnivores are among the most threatened species of the world (Ripple *et al.*, 2014), with especially felids experiencing a significant contraction from their historical range (Wolf and Ripple, 2017). They play an important role in maintaining ecological balance as apex predators (Terborgh *et al.*, 2001) and are under severe threat due to habitat fragmentation, habitat loss and isolation, reduction in genetic diversity, prey depletion and poaching (Morell, 2007; Walston *et al.*, 2010). In addition, their biological traits, e.g. solitary life, and large individual home ranges render them vulnerable to threats associated with increasing human population densities (Cardillo *et al.*, 2004).

The tiger (*Panthera tigris*) is an endangered species that has lost >95% of its global historical home range (Wolf and Ripple, 2017), and its extant population now exists in fragmented habitats across its former area (Ranganathan *et al.*, 2008). Despite steep declines in population size and habitat, the Indian subcontinent remains a key area for tiger conservation as it harbours around 60% of the current global free-roaming tiger population (Mondol *et al.*, 2009). However, tigers continue to suffer from several anthropogenic threats like poaching, habitat loss and fragmentation (Jhala *et al*. 2008; Ranganathan *et al.*, 2008; Mondol *et al.*, 2013; Goodrich *et al.*, 2015). Consequentially, most of the tiger populations now occur in protected areas (PAs), which are pockets of habitats embedded in human-dominated landscapes and are usually not big enough to hold demographically viable populations by themselves (Ranganathan *et al.*, 2008). Thus, the status of the tiger remains threatened despite of the various conservation efforts. Recent conservation management strategies focus on landscapes including more than one metapopulation (Wikramanayake *et al.*, 2004). The outcome of these efforts has led to the identification of ‘Tiger Conservation Landscapes’, which include interconnected PAs by corridors that could potentially support viable populations (Dinerstein *et al.*, 2007; Sanderson *et al.*, 2010; Joshi *et al.*, 2013).

At present, India harbours over 1.21 billion people (Census of India 2018) and it is projected to even increase to 1.4 billion people by 2022 (World Population Prospects, UN, 2015). The country has still 21% forest cover (Reddy *et al.*, 2013), but only 5% of this land is protected, which largely resides in human-dominated landscapes. Rural households in India depend on locally available resources from the forests for their domestic needs, which include fuelwood, grazing ground for animals and other non-timber forest produce (Hussain *et al.*, 2016). Human presence usually disturbs wildlife, causing animals to focus on people avoidance, thereby potentially reducing reproductive success (Ciuti *et al.*, 2012). In addition, ecotourism in PAs has substantially increased over the last decade (Reed and Merenlender, 2008), and although these activities generate revenue and provision employment for local communities (Buultjens *et al.*, 2005), there have been concerns over the impact of tourism on conservation goals (Ranaweerage *et al.*, 2015). It has been shown that human disturbance, as well as tourism pressures, can act as potential stressors for wildlife, evoking physiological stress and fitness responses (Zwijacz-Kozica *et al.*, 2012; Hadinger *et al.*, 2015; Coetzee and Chown, 2016).

A perceived stressor induces the release of glucocorticoids, which enables the animal to cope and restore homeostasis. A short-term release of glucocorticoids usually enhances fitness benefits via energy mobilization, but chronically elevated glucocorticoid levels can negatively impact many physiological processes including growth, reproductive success, immunosuppression and muscular atrophy (Wingfield *et al.*, 1998; Charbonnel *et al.*, 2008; French *et al.*, 2010; Hadinger *et al.*, 2015). Prolonged anthropogenic disturbance has been shown to increase glucocorticoid levels in many wild species across taxa including amphibians (Janin *et al.*, 2011), reptiles (Knapp *et al.*, 2013), birds (Wasser *et al.*, 1997) and mammals (Creel *et al.*, 2002, 2013; Van Meter *et al.*, 2009). Prolonged stress can directly affect behaviour, fitness and reproductive success (Young *et al.*, 2006; Kumar *et al.*, 2014) and consequently may lead to an overall population decline (Strasser and Heath, 2013). Although reproduction of both sexes can be affected by stress, females tend to be more sensitive in some species, such as Sumatran and Bengal tiger (Barja *et al.*, 2007, 2011; Narayan *et al.*, 2013a, 2013b; Bhattacharjee *et al.*, 2015). Given the challenges mentioned above, it is crucial to monitor the effect of long-term and persistent anthropogenic mediated stressors in iconic and keystone wildlife species like the tiger.

This study examined the relationship between anthropogenic disturbance and physiological stress levels in two tiger populations in central India by assessing faecal glucocorticoid metabolite (fGCM) concentrations of individual tigers and the status of anthropogenic disturbance of the related reserves during tourism and non-tourism periods. We also examined the differential sensitivity of male and female tigers with reference to anthropogenic disturbance. We hypothesized that individuals in areas under high tourism pressure and proximity to human settlements would perceive more stress reflected in higher fGCM concentrations, with higher stress steroid level found in females compared to males.

## Materials and methods

### Study sites

**Figure 1:**
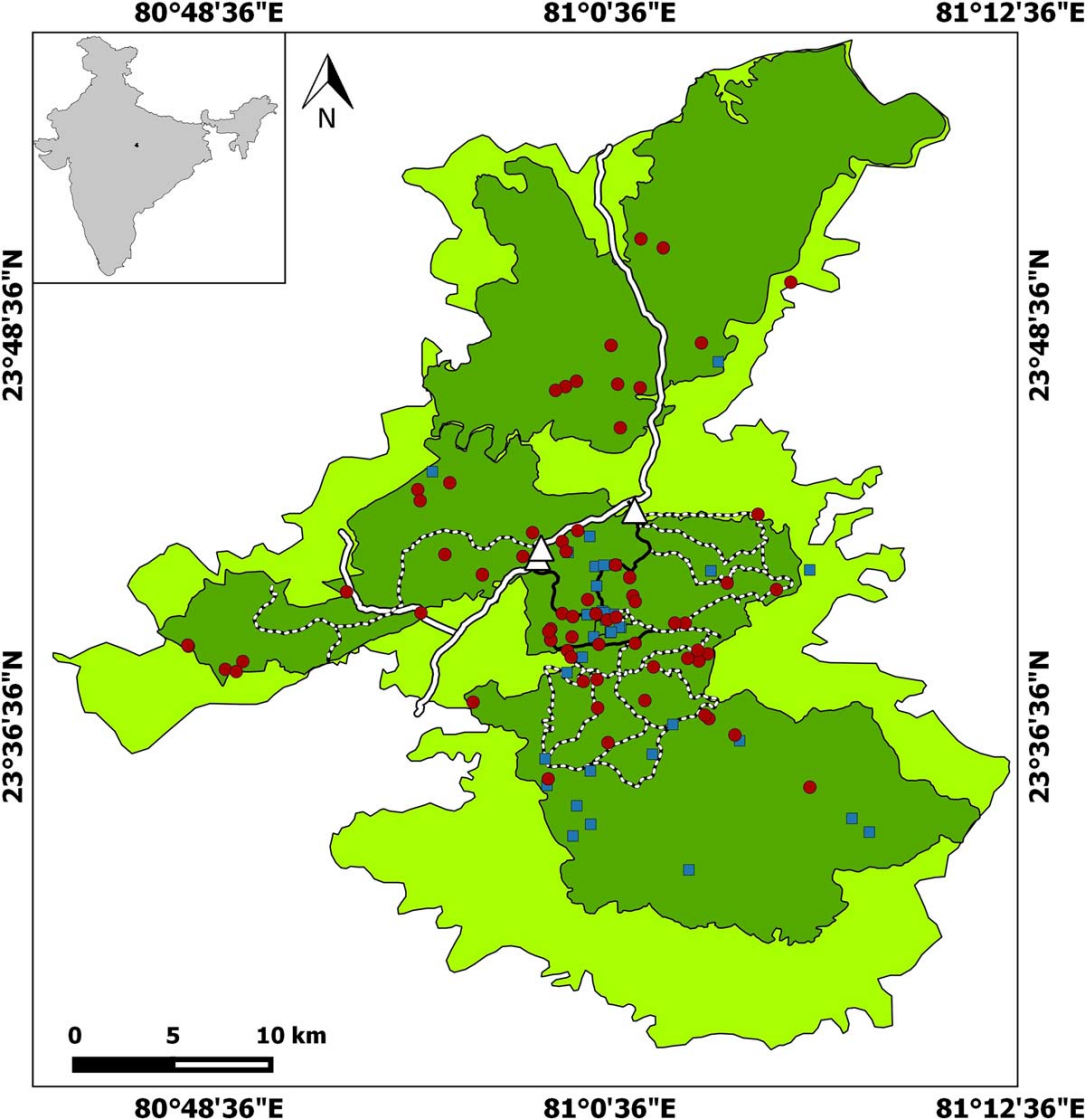
Map showing BTR sample locations and tourist routes (solid black line, ≥51 vehicles per day; dotted line, <50 vehicles per day; white thick line, state highway road). Red round represents samples collected during tourism period, while the blue square represents sample collected during the non-tourism period. Dark green colour in the map is core zone while florescent colour is buffer zone. The white triangle is the entry point to the park.

**Figure 2:**
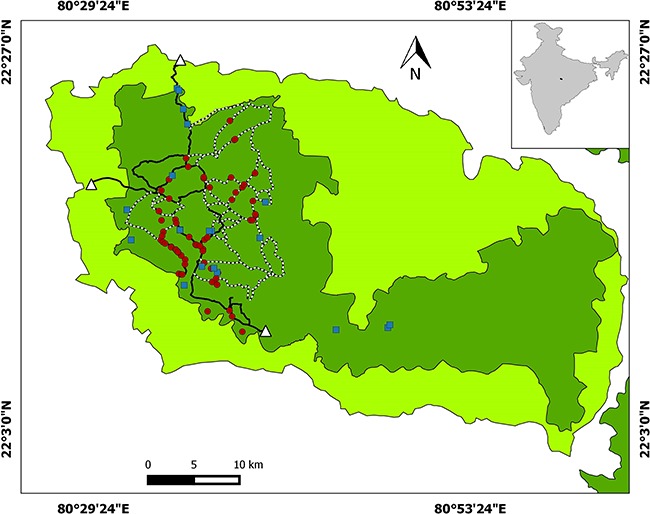
Map showing KTR sample locations and tourist routes (solid line, ≥51 vehicles per day; dotted line, < 50 vehicles per day). Red round represents samples collected during tourism period while the blue square represents sample collected during the non-tourism period. Dark green colour in the map is core zone while fluorescent colour is buffer zone. The white triangle is the entry point to the park.

Both study sites, Bandhavgarh Tiger Reserve (BTR) and Kanha Tiger Reserve (KTR) (Figs 1 and 2, respectively), are situated in Madhya Pradesh state of central India, which is regarded as a global priority tiger conservation landscape (Sanderson *et al.*, 2010). Both parks have a similar habitat of primarily tropical moist deciduous sal (*Shorea robusta*) forests. The surrounding landscape is a matrix of forest and human land use habitats. The average rainfall is 1173 mm, most of which occurs between July and October (Sankar *et al.*, 2013).

The multi-use buffer zone of KTR spans around 1134 km^2^ where human habitation and other anthropogenic activities like cattle grazing are allowed. The buffer zone surrounds a 917 km^2^ core area of where no anthropogenic activity is permitted except tourism. The reserve consists of two conservation units: Kanha National Park and Phen Wildlife Sanctuary. The core zone of Kanha is inhabited by more than 8000 humans and approximately 7000 cattle. The buffer zone is experiencing further strong anthropogenic pressure by supporting around 129 300 people and more than 85 000 cattle (Miller *et al.*, 2015).

The BTR lies on the north-eastern border of the Madhya Pradesh state and is situated north of KTR. The BTR consists of two PAs: Bandhavgarh National Park and Panphata Wildlife Sanctuary, with a total area of 1598 km^2^ (716 km^2^ of core area surrounded by 820 km^2^ buffer area). There are 15 villages located inside BTR, harbouring a human population of over 60 000 with more than 110 000 livestock (Sankar *et al.*, 2013).

Both reserves, KTR and BTR, support large tiger populations of over 60 individuals each (Dutta *et al.*, 2016), which make them major tiger tourism spots attracting huge numbers of tourists each year. We acquired data from the forest department on the number of tourists and related vehicles entering the park (permission from the Principal Chief Conservator of Forests, Madhya Pradesh letter Reference No. 7616, dated 12 October 2014). The forest department monitors the number of tourists and vehicles at all entry points and permits a maximum of 50 vehicles per day (morning and evening sessions combined) from each entry point. We estimated the mean number of people visited and the mean number of vehicle entered into the parks based on the data obtained from Forest department.

### Faecal sample collection

Samples were collected from both reserves between January and March (tourism period) as well as in September (non-tourism period) in 2015. We surveyed pre-existing forest roads and trails to collect faecal material, and only fresh samples, ~1 day old (based on the outline shape, moisture content, smell and insect activity), were collected. Collected faecal samples were split into two portions, for hormone analysis and DNA profiling, respectively. For DNA profiling, samples were sprayed with ethanol and dried using a hot air blower on the same day of collection and then stored with silica beads in zip lock bags. For hormone analysis, the collected samples were partially extracted in the field and then transported to the laboratory (Council for Scientific and Industrial Research—Centre for Cellular and Molecular Biology) for further processing. Each road/trail was sampled only once in 3 days to avoid collection of faecal samples from the same individual and to maximize area coverage. Geographic location using GPS and other information such as signs of the presence of livestock and villagers, woodcutting and lopping and tourist vehicle per day on the particular trail/road were recorded for each sample.

### DNA extraction

Faecal material was dried overnight in a hot air oven at 50°C to remove any moisture. DNA extraction was carried out in separate, pre-PCR (Polymerase chain reaction) laboratory space, in a set of *n* = 11 samples along with an extraction control to monitor the risk of contamination. Faecal surface material was taken for isolation of DNA. Genomic DNA was subsequently extracted using a Qiagen stool kit following the manufacturer’s protocol. The extracted DNA was stored in elution buffer, and DNA quantification was conducted using a NanoDrop spectrometer.

### Species confirmation and identification of the sexes

Three tiger-specific mitochondrial markers have been used to ascertain the species identity of collected faecal material. Two tiger-specific primers (Tig490 and Tig509) amplify short fragments from two regions of the NADH5 sub-unit (Mukherjee *et al.*, 2007) and one primer (TIF/TIR) amplifying a short region of the mitochondrial cytochrome b gene were used for species identification (Bhagavatula and Singh, 2006).

Sex was identified using two sets of markers previously designed and standardized for felids (Pilgrim *et al.*, 2005). The two primers amplify the zinc finger (Znf) and amylogenin (Amg) domain on the sex chromosomes, respectively. PCRs were carried out using the protocol as described in the study by Pilgrim *et al.* (2005). The total reaction volume was 15 μl with 1× BSA, 1× PCR buffer, 0.25 mM of each dNTP, 2.5 mM MgCl2, 0.25 μM each of forward and reverse primers and 0.75 units of Taq polymerase (ExTaq HS DNA polymerase, Takara Bio Inc.). All PCR reactions were carried out with multiple negative controls. Pre- and post-PCR work was carried out at separate places. Visualization of PCR products was done on a 2% agarose gel.

### Faecal steroid extraction and analysis

Faecal steroid extraction and fGCM quantification was carried out using earlier described procedures (Umapathy *et al.*, 2013; Bhattacharjee *et al.*, 2015). About 0.2 g of dried and pulverized faecal material was boiled in 5 ml of 90% of ethanol for 20 min. After centrifugation at 500 g for 10 min, the supernatant was decanted and the pellet re-suspended in 5 ml of 90% ethanol, vortexed for 1 min and again centrifuged to recover the supernatant. Both supernatants were combined, dried in an oven at 40°C, re-suspended in 1 ml of absolute methanol by vortexing for 1 min and then stored at −20°C until further processing.

We determined fGCM concentrations in faecal extracts using a cortisol enzyme immunoassay (EIA) (Dr Coralie Munro, University of California, Davis). The cortisol EIA has shown to provide reliable information on adrenocortical function in tigers (Bhattacharjee *et al.*, 2015). The assay was carried out as previously described in Kumar *et al.* (2014) and Bhattacharjee *et al.* (2015). Serial dilution of pooled faecal extracts of tigers gave parallel displacement curves to the respective standard curves of the cortisol assay. Assay sensitivity at 90% binding was 0.195 ng/g dried faecal matter. Intra- and inter-assay coefficients of variation determined by repeated measurements of quality controls were 4.25% and 7.49%, respectively.

### Estimating anthropogenic disturbance

To estimate the anthropogenic disturbance due to tourism, we obtained information from the forest department about the number of vehicles entering the park daily. In both reserves, there are multiple routes and entry points for tourist vehicles. Sample locations were characterized as high, moderate, less and no disturbance based on the presence of livestock and villagers, wood cutting and lopping and vehicular movements (Bhattacharjee *et al.*, 2015). In BTR, the number samples collected were 40 (from no or less disturbance area), 19 (low disturbance), 35 (moderate disturbance) and 20 (high disturbance), while in KTR, the number samples were 21, 8, 45 and 17 from no or less, low, moderate and high disturbance areas, respectively.

### Data analysis

fGCM concentrations are given as ng/g faecal dry weight (DW) with respective hormone values being presented as mean and standard error. Since our data were not normally distributed, we used non-parametric tests for analyses. Mann–Whitney *U* test (M–W test) was used to test for differences in fGCM concentrations between the two seasons (tourism, October–June; non-tourism, July–September) as well as the two reserves (BTR and KTR). A generalized linear model (GLM) was used to examine variations in fGCM concentrations with reference to various factors (sex, season, anthropogenic disturbance level and location—buffer and core) as the explanatory variables, which included both continuous and categorical data, and the response variable was continuous data. Pearson correlation was used to examine the relationship between mean number of vehicles traversed per day and fGCM concentration of tigers in that location. A data analysis was carried out using SPSS ver 17.1.

## Results

### Tourists and vehicles

In total, 244 179 people visited both BTR (106 535) and KTR (137 644) during the 9 months of tourism season (October 2014–June 2015), with an average of 395 people/day in BTR and 509 people/day in KTR. To travel inside the parks, 51 695 vehicles have been used in total (BTR, 23 011; KTR, 28 684) during the study with an average of 85 and 106 vehicles/day in BTR and KTR, respectively.

### Sample collection and DNA profiling

We collected 341 suspected tiger faecal samples in total, of which 206 samples (BTR, 114; KTR, 92) were identified to be samples from tigers. Of the 114 samples collected from BTR, 91 (41 from males; 50 from females) were collected between January and March (tourism period) and 23 (11 from males; 12 from females) samples during September 2015 (non-tourism period). In KTR, 75 (43 from males; 32 from females) samples were collected between January and March) and 17 (13 from males; 4 from females) samples during September 2015.

### GCM concentration in faecal samples

Overall mean fGCM concentrations of tigers roaming at BTR (51.45 ± 4.75 ng/g DW) and KTR (56.46 ± 6.6 ng/g DW) were not significantly different (M–W test, *n* = 206, *P* = 0.87).

**Figure 3:**
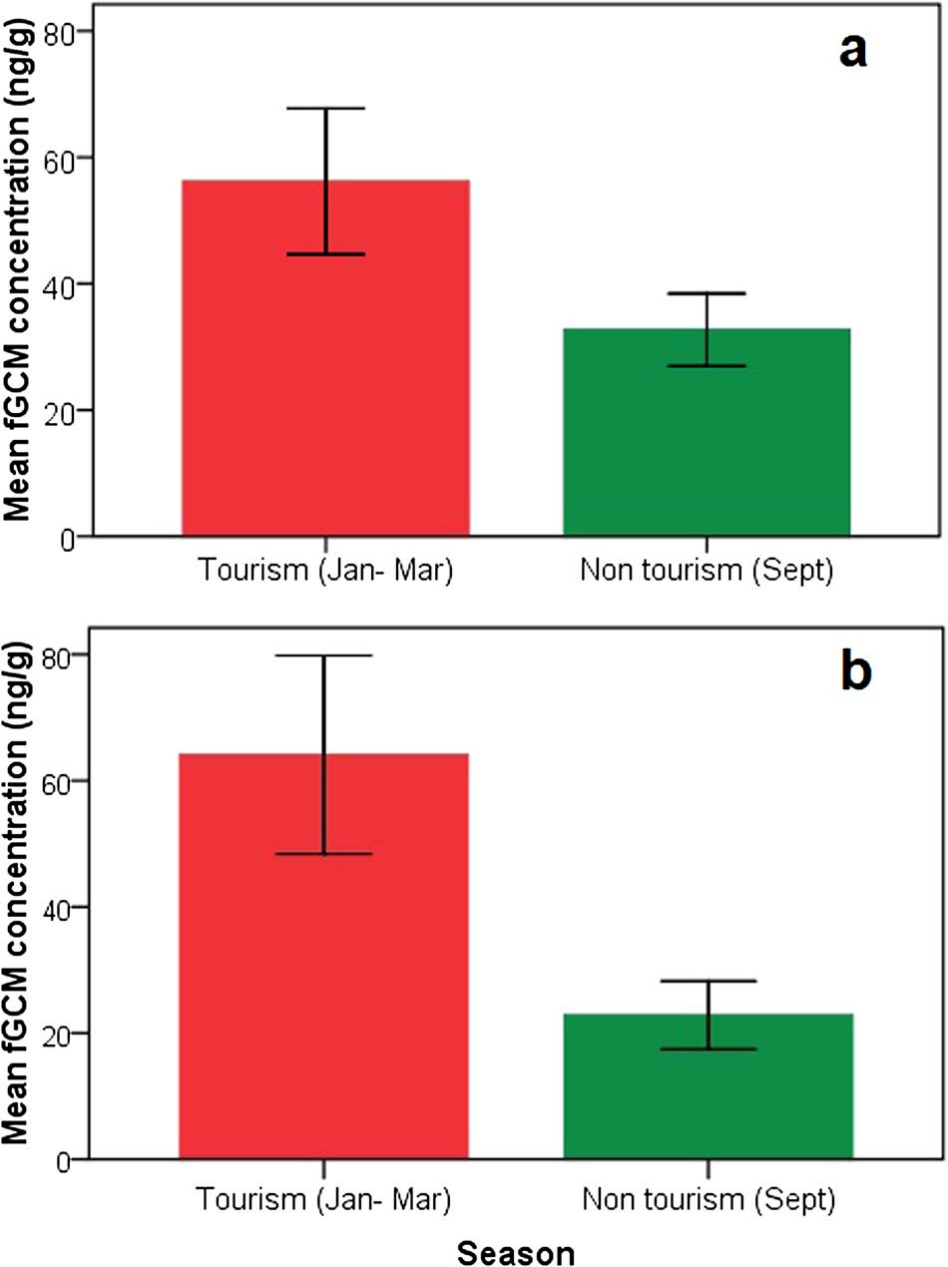
(a and b) Mean (±SEM) fGCM concentrations in tigers during tourism period (January–March) and non-tourism (September) in BTR and KTR

**Figure 4:**
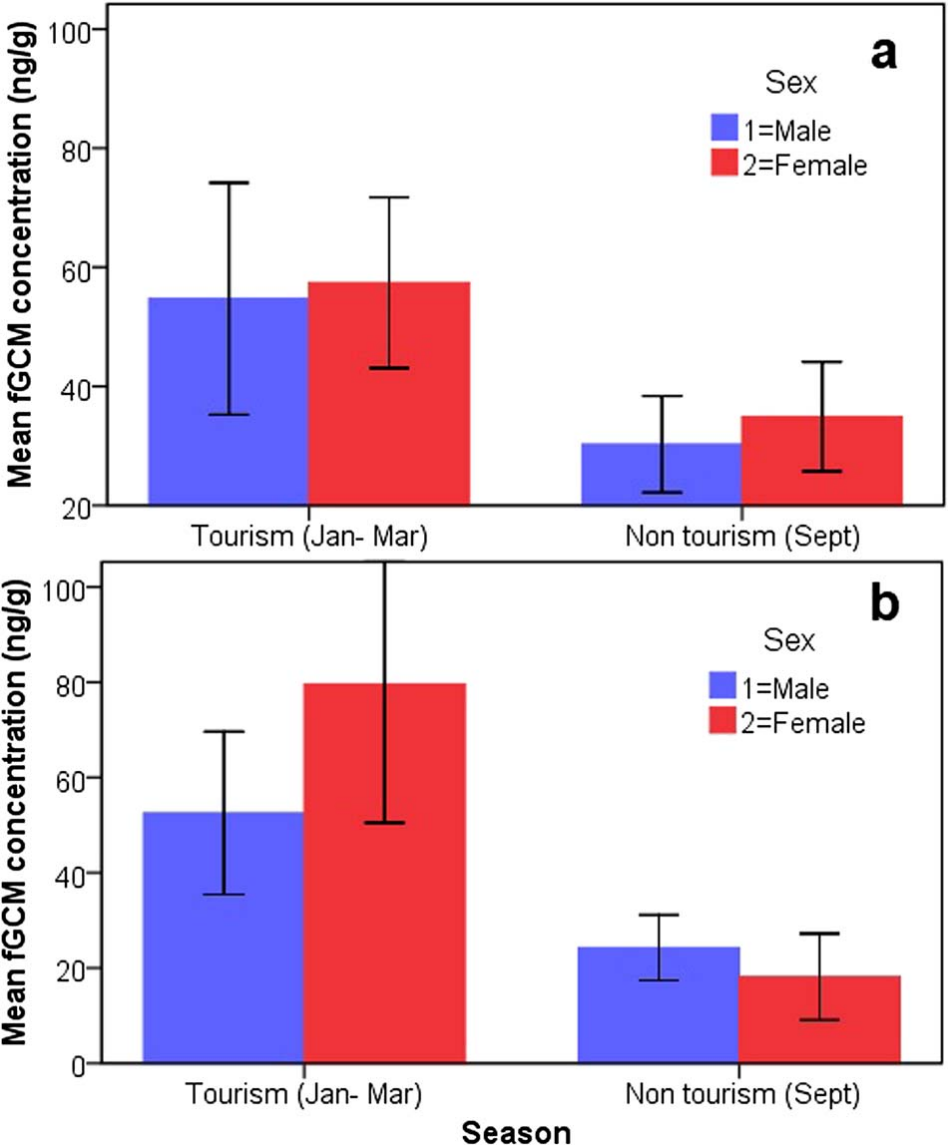
(a and b) Mean (±SEM) fGCM concentrations between tourism and non-tourism seasons among male and female in BTR (a) and KTR (b)

**Figure 5:**
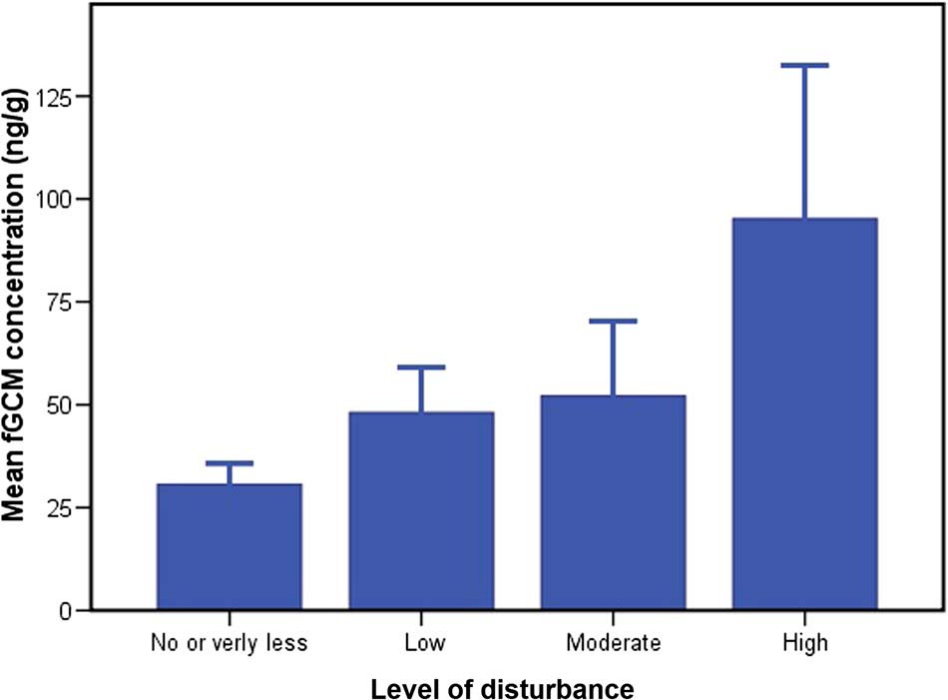
Mean (±SEM) fGCM concentrations in tigers with reference to disturbance level including high vehicular movement in BTR

We found significantly higher fGCM concentrations in tigers at BTR during the tourism period (56.47 ± 5.81 ng/g DW, *n* = 91) compared to the non-tourism period (32.69 ± 2.76 ng/g DW, *n* = 23; M–W test, *n* = 114, *P* = 0.001; Fig. 3a). Similarly, fGCM concentrations showed a positive correlation with the number of vehicles visited per day during the tourism period (*r* = 0.34; *P* = 0.001; *n* = 91). There were no significant differences in fGCM concentrations between the sexes during tourism and non-tourism period (M–W test, *n* = 91, *P* = 0.15 and *n* = 23, *P* = 0.56, respectively; Fig. 4a). GLM results showed that fGCM concentrations are significantly influenced by tourism season (*F*_1_ = 4.710; *P* = 0.032), number of vehicles (*F*_4_ = 3.97; *P* = 0.010) and disturbance level (*F*_3_ = 6.62; *P* = 0.0001; Fig. 5). Sex and sample location (core and buffer) did not influence fGCM concentrations determined during the study period (GLM *F_1_* = 0.13; *P* = 0.60 and *F_1_* = 0.033; *P* = 0.75, respectively).

Similarly, we found significantly higher fGCM concentrations in tigers at KTR during the tourism period (64.09 ± 7.88 ng/g DW; *n* = 92) compared to the non-tourism period (22.82 ± 2.54 ng/g DW; *n* = 17; M–W test, *n* = 109, *P* = 0.001; Fig. 3b). For the tourism as well as non-tourism period, no significant differences in fGCM concentrations were found between the sexes (M–W test, *n* = 92, *P* = 0.24 and *n* = 17, *P* = 0.29, respectively; Fig. 4b). A GLM analysis showed that fGCM concentrations are significantly influenced by tourism (*F_1_ =* 10.07; *P* = 0.001) but not by sex (*F_1_* = 0.130; *P* = 0.27), disturbance level (*F_3_* = 0.011; *P* = 0.57), number of vehicles (*F_3_* = 0.09; *P* = 0.62) or location (*F_1_* = 0.044; *P* = 0.85).

## Discussion

Understanding the impact of anthropogenic stressors on tiger populations can provide valuable information for optimizing conservation and management strategies. Our study showed that wildlife tourism can cause distinct physiological stress in tigers in PAs. A significant positive correlation was observed between fGCM concentrations and the number of vehicles visiting BTR. These results are concordant with results of other studies on various wildlife species. Bhattacharjee *et al.* (2015) demonstrated that reintroduced tigers show high fGCM levels when challenged by anthropogenic disturbance such as traffic, human encounters and manned livestock. Other researchers have demonstrated that the use of snowmobiles increased fGCM levels in elk (*Cervus elephus*) and wolves (*Canis lupus*) (Creel et al., 2002). Similarly, increasing fGCM concentrations have been found in relation to anthropogenic disturbance, e.g. for African lions (*Panthera leo*) roaming within a human-dominated buffer zone (Creel *et al.*, 2013), spotted hyenas (*Crocuta crocuta*) occurring in disturbed areas of a National Reserve (Van Meter *et al.*, 2009) or free-roaming European pine martens (*Martens martens*) occurring near tourist areas in a natural park (Barja *et al.*, 2007).

Perception of prolonged stress is known to affect survival and reproduction by influencing the immune system and increase susceptibility to diseases (Munck *et al.*, 1984, Arlettaz et al. 2007). One of our previous studies has demonstrated that recently introduced tigers failed to reproduce effectively presumably due to high levels of stress caused by high anthropogenic disturbance (Bhattacharjee *et al.*, 2015). Although some individuals might adjust to the presence of humans, the overall pattern of increased fGCM concentrations found in this study clearly indicate that tourism can elevate physiological stress in tigers, which may affect the reproductive potential on a population level. Although we cannot exclude the possibility of a potential impact of reproductive state or age on fGCM output, our study did not find any significant difference in fGCM concentrations between the sexes. Thus, our findings are more likely related to the anthropogenic disturbances described rather than potential sex- or reproductive status-biassed stress (Creel *et al.*, 2013; Narayan *et al.*, 2013a; Webster *et al.,* 2018).

Current guidelines from the National Tiger Conservation Authority (NTCA) limit tourism activities to 20% of the core area and restricting vehicle access to 40 cars per day in an Indian reserve (NTCA management plan 2010). Although we were unable to exactly estimate the percentage of core area used for tourism at our study sites; it seems often impacted beyond the recommended 20% of the core area in BTR (see Fig. 1), and the number of vehicles entering BTR also exceeds the recommended number according to a respective management document (National Tiger Conservation Authority, 2010). Similarly, KTR management has permitted an average of 106 vehicles (officially recorded) per day against the 40 car per day recommended by the NTCA. Furthermore, recommended distances between vehicles is not often followed during a tiger sighting, which leads to an over-crowding of vehicles around the animal (pers. obs.). This behaviour might directly affect the territorial and mating behaviour of tigers, resulting in an overall lower reproductive success due to increased stress e.g. for tigers (Bhattacharjee *et al.*, 2015) and wild cats (Piñeiro *et al.*, 2012). Since carnivores occur in low densities, changes in reproductive success and survival rate of especially adult females can severely affect the sustainability of isolated populations (Knight and Eberhardt, 1985; Smith and Mcdougal, 1991; Kerley *et al.*, 2002). Overall, such disturbance can have severe implications on the survival of wildlife populations, especially of tigers, which are facing the multi-dimensional threat to their existence in an increasing human-modified landscape.

We demonstrate that tourism and thus anthropogenic disturbance are correlated with fGCM concentrations of tigers in both monitored reserves. Although both reserves experience a similar tourism pressure, the stronger correlation found in BTR might be attributed to the comparatively higher number of human settlements and cattle densities in and around the reserve. Since the tigers at BTR are genetically less connected to other populations as the ones at KTR (Yumnam *et al.*, 2014; Thatte *et al.*, 2018), conservation efforts should even focus on the BTR population. However, as our study only provides a snapshot of the effects of anthropogenic disturbance on tiger, long-term, individual-based studies with greater spatial and temporal sampling would be crucial to better understand the adverse effects of anthropogenic stressors on the physiology of this keystone species.

Our management recommendations include strict regulation of vehicular traffic and number of tourist vehicle, shifting of artificial waterholes away from tourist roads and reducing other anthropogenic disturbance, including relocation of villages from the core area of a tiger reserve.
